# Transition from Vehicular to Structural Ionic Transport in Electrified Alkali Aqueous Solutions

**DOI:** 10.1021/acs.jpcb.5c07449

**Published:** 2026-03-02

**Authors:** Kit Joll, Philipp Schienbein, Kevin M. Rosso, Jochen Blumberger

**Affiliations:** † Department of Physics and Astronomy and Thomas Young Centre, 4919 University College London, London WC1E 6BT, U.K.; ‡ Lehrstuhl für Theoretische Chemie II, Ruhr-Universität Bochum, 44780 Bochum, Germany; § Research Center Chemical Sciences and Sustainability, Research Alliance Ruhr, 44780 Bochum, Germany; ∥ 6865 Pacific Northwest National Laboratory, Richland, Washington 99354, United States

## Abstract

A molecular understanding of the solvation and dynamics of ions under static electric fields is crucial for modeling a wide range of natural and technological processes. Yet, traditional simulation methods suffer from a trade-off that has to be made between accuracy and statistical convergence. To bridge this gap, herein, we extend our recently introduced perturbed neural network potential molecular dynamics (PNNP MD) approach to investigate the solvation structures and ionic transport mechanisms of electrified alkali cationic solutions. We obtain ionic conductivities for Li^+^, Na^+^, and Cs^+^ from the field dependence of the ionic current density in good agreement with experiment. Surprisingly, the migration mechanism is found to be strikingly different for the three ions, despite their similar ionic conductivities. While Li^+^ conducts predominantly through *vehicular* migration of a stable 4-fold coordinated ion at all field strengths, Cs^+^ conducts strictly through a *structural* diffusion mechanism, where 9–12 transient first shell water coordination bonds are continuously broken and reformed. Notably, aqueous Na^+^ emerges as a “Goldilocks” ion: its ion–water interactions are strong enough to maintain distinct 5–6-fold coordination shells at zero field (unlike Cs^+^) yet labile enough to be strongly perturbed by electric fields (unlike Li^+^). As a consequence, we observe an electric-field-induced transition from vehicular to structural ionic transport for Na^+^ that is accompanied by a marked increase in ionic current density. Our results imply that the conductance mechanism of ions with moderate ion-solvent interactions can be effectively tuned by external electric fields.

## Graphical abstract

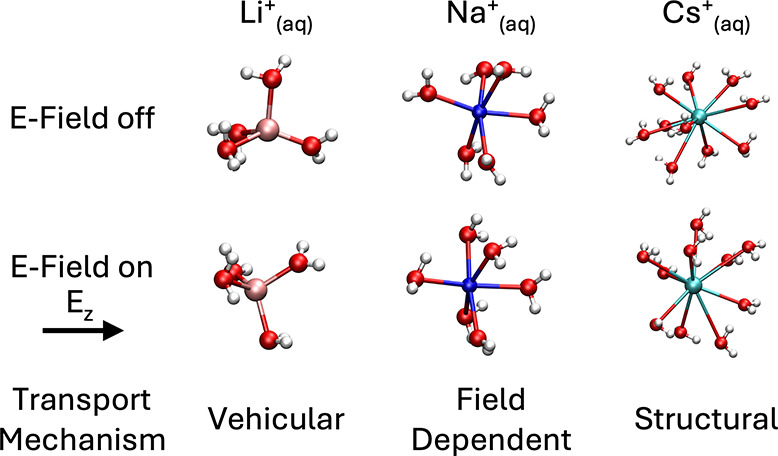

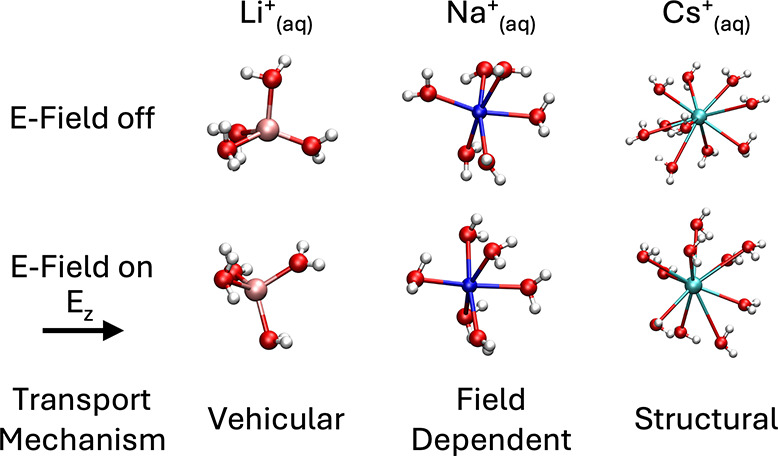

## 1 Introduction

Hydrated ions underpin a plethora of chemical and biological processes, ranging from charge transport in energy storage devices to ionic conduction in biological channels and redox reactivity at electrified interfaces.^1–4^ The experimental investigation of aqueous ionic solutions has a long history with techniques such as neutron and X-ray scattering,^5,6^ extended X-ray absorption fine structure (EXAFS),^7^ ultrafast infrared and Raman spectroscopy,^8–10^ and ionic conductivity measurements,^11^ providing valuable insight into coordination numbers, hydration-shell exchange rates, ionic conductivity, and reactivity.^3,4,12–22^

In realistic environments such as electrochemical interfaces or biological systems, aqueous ions rarely experience zero-field conditions. Static electric fields of order 0.1 V Å^–1^ arise naturally near charged electrodes, within double layers, and in confined electrolytes.^1,2,23^ Such fields couple strongly to the molecular dipole moments of water molecules and the charge density of solvated ions, driving reorientation of hydrogen-bond networks, perturbing ion–water coordination thermodynamics, and modifying ligand exchange kinetics. As a consequence, electric fields can alter the fundamental mechanism of ionic transport: ions may migrate in a *vehicular* manner, dragging most of their hydration shell with them, or via *structural* diffusion, hopping between successive solvation sites.^24,25^ Capturing these different transport scenarios and possible field-induced transitions between them is key to understanding conductivity and selectivity in electrified environments. However, direct experimental observation of such microscopic processes is challenging, motivating a central role for molecular simulations.

Previous simulation studies with external electric fields have typically employed empirical force fields.^18,26–30^ These studies investigated how water dipoles align with external fields, the ionic conductivity of given solutions, and how electric double layers form. However, force field models, even in the absence of external fields, are often not accurate enough to account for the true complexity of ionic solvation and may yield qualitatively incorrect results. This is particularly problematic for soft ions where polarization and charge transfer effects are significant but are usually neglected in force field models.^21^ Ab initio molecular dynamics (AIMD) has been applied to model liquid water and electrolyte systems at finite electric fields using the Berry-phase or related approaches,^31–33^ providing valuable insight into the impact of the field on the structure and dynamics of these systems. However, AIMD is too computationally demanding to sample the long trajectories required to converge ionic transport properties across a wide range of field strengths.

Over the past decade, machine learned potentials^19,20,34–44^ and many-body potentials^19,20,45^ have emerged as a promising avenue, retaining the accuracy of the underlying electronic structure methods, but being several orders of magnitude faster, enabling the simulation of complex interactions and dynamics over sufficiently long time scales. Since then, a variety of different investigations into ionic aqueous systems have been conducted using machine learning potential-based molecular dynamics (MLMD)^16,46–48^ that are simply unfeasible with direct AIMD approaches. For instance, Shao et al. simulated the ionic conductivity of aqueous NaOH solutions over a wide range of temperatures and concentrations;^46^ O’Neill et al. simulated NaCl dissolution for over 300 ns^47^ and investigated ion pairing in NaCl solutions with ML potentials trained to MP2 and RPA electronic structure calculations; and Joll et al. succeeded in converging the potential of mean force for chemisorption of Fe^2+^ on hematite (001) using multinanosecond MLMD trained to hybrid DFT electronic structure calculations.^16^

To enable MLMD simulations of systems interacting with external electric fields, we have recently introduced the perturbed neural network potential (PNNP) molecular dynamics approach (PNNP MD).^49^ In PNNP MD, the total force on each atom is partitioned into an unperturbed component, captured by a committee of high-dimensional neural network potentials (c-NNP),^34,50,51^ and a perturbed component arising from the interaction of the system with an external static or time-dependent electric field, which is described by an atomic polar tensor neural network (APTNN).^49,52,53^ In recent work, we demonstrated that this simple perturbative ML scheme captures the interaction of pure liquid water with surprisingly large external fields at a negligible loss of accuracy when compared to DFT-level reference calculations, resulting in a computationally efficient and scalable finite-field molecular simulation method. We also demonstrated that PNNP MD simulates dipole relaxation time, the dielectric constant, and field-dependent infrared spectra of liquid water in very good agreement with experimental data.^49^

In this work, we extend PNNP MD to the simulation of electrified alkali cationic solutions, specifically Li_(aq)_^+^, Na_(aq)_^+^, and Cs_(aq)_^+^ solutions, up to field strengths that are typical for energy storage devices, ∼0.2 V Å^–1^. We simulate a single cation in solution without the presence of a counterion, which allows us to study the impact of external electric fields on the solvation structure and ionic conductivity in isolation from the impact of other effects such as ion pairing or ion–ion correlations. We choose Li^+^, Na^+^, and Cs^+^ because they display a seemingly counterintuitive experimental trend: the heaviest ion has the largest diffusion constant (Cs^+^) and the lightest ion the lowest (Li^+^). This is usually explained by the higher charge density and hence larger solvation shell and higher effective mass of the lighter ion compared to those of the heavier ion. Our simulations show that this picture culminates in two qualitatively different transport mechanisms for the two ions: while Li^+^ migrates predominantly as a stable tetra-aquo ion via a vehicular mechanism, Cs^+^ migrates exclusively as a bare ion via a structural (or hopping) mechanism. The transport mechanism of these ions is robust and remains unchanged, even when strong external electric fields are applied. On the other hand, Na^+^ undergoes a field-induced transition from vehicular to structural transport accompanied by a conductivity increase at high fields. These results highlight the impact of field-driven reorganization of hydration shells on ionic conduction and demonstrate the power of PNNP MD in understanding finite-field effects on ionic conduction in aqueous media.

## 2 Methods

### 2.1 PNNP MD

The PNNP approach has been introduced and discussed in detail in ref 49. In this method, the interaction of the atomistic system with a homogeneous external electric field **ε** is treated perturbatively:^18,23,54,55^

1 $$H_{\varepsilon }\left(r^{N},p^{N}\right)=H_{0}\left(r^{N},p^{N}\right)+H_{p}\left(r^{N}\right)$$

where $H_{0}\left(r^{N},p^{N}\right)$ is the total unperturbed Hamiltonian comprising the kinetic energy of the *N* nuclei with momenta **p** ^*N*^ and the electronic potential energy depending on all nuclear positions **r** ^*N*^, $H_{0}\left(r^{N},p^{N}\right)=E_{kin}\left(p^{N}\right)+E_{pot}\left(r^{N}\right)$ , and $H_{p}\left(r^{N}\right)$ is the perturbation induced by the homogeneous electric field **ε** which is truncated at first order in the field,

2 $$H_{p}\left(r^{N}\right)=-\varepsilon \cdot M\left(r^{N}\right)$$

with **M**(**r** ^*N*^) being the total dipole moment of the system at zero field. The total force acting on atom *i* is given by the unperturbed and perturbed contributions, *F*_*i*,η_^0^ and *F*_*i*,η_^p^, respectively,

3 $$F_{i,\eta }=F_{i,\eta }^{0}+F_{i,\eta }^{p}$$

4 $$F_{i,\eta }^{0}=-\frac{\partial E_{pot}\left(r^{N}\right)}{\partial r_{i,\eta }}$$

5 $$F_{i,\eta }^{p}=\sum_{\zeta }\frac{\partial M_{\zeta }}{\partial r_{i,\eta }}\varepsilon_{\zeta }=\sum_{\zeta }P_{i,\eta ,\zeta }\varepsilon_{\zeta }$$

The diagonal elements of the APT are related to the Born effective charge of atom *i*, $q_{i}=\left(1/3\right)tr\left(P_{i}\right)$ . In our approach, we train two ML models: a committee^51^ of second-generation high-dimensional neural network potentials^34,50^ (c-NNP) to model the unperturbed forces, eq 4, and an E(3)-equivariant graph neural network to model the APT (APTNN), via eq 9, which gives the perturbed atomic forces, eq 5. Note that the PNNP approach is compatible with any chosen potential to model the unperturbed forces. The total forces in eq 3 are used to carry out MD simulations with a finite electric field, which is referred to as a PNNP MD simulation.

### 2.2 c-NNP Training

The training of the c-NNP was split into two steps: in the first step, an initial c-NNP is generated by learning DFT energies and forces of reference configurations obtained from AIMD simulations of the aqueous ions, following the procedure outlined in refs 51 and 56 . Details on the DFT calculations and the AIMD simulations are given further below. In the second step, the initial c-NNP was used to run c-NNP MD simulations on 5 systems containing 1 ion and 64, 128, 256, 512, and 1024 water molecules. Details of the generation of the simulation cells are given further below. The aim of this second stage was to explore phase space to generate and learn configurations that have not been sampled on the time scale of AIMD. Each system was equilibrated for 20 ps to 300 K in the *NVT* ensemble (Nosé–Hoover thermostat with a chain length of 3, global region, and a time constant of 50 fs^57,58^ ) using the initial c-NNP. During the subsequent active learning stage, the time constant was increased to 200 fs. For each system, c-NNP simulations were run until the network failed, as indicated by a peak in the committee variance. Then, for the 64 and 128 water molecule systems, DFT energies and forces were calculated on structures at the onset of the rise in committee variance for between 10 and 20 of the highest variance structures. This DFT data was then added to the training set, and the c-NNP was retrained. This second step was repeated until the c-NNP was able to simulate 1 ns for each of the 5 systems. The final c-NNPs were trained on 500 structures for Na_(aq)_^+^ and Li_(aq)_^+^ and 600 structures for Cs_(aq)_^+^. The c-NNP was trained using the n2p2 package, and second-generation c-NNPs were employed.^34,50,59^ The standard set of symmetry functions was used, with a cutoff of 6 Å,^51,60^ along with two hidden layers containing 25 nodes, respectively. The hidden layers were activated using the hyperbolic tangent function, and the output layer utilized a linear activation function. The weights and biases were randomly initialized for each of the 8 committee members and then optimized using the Kalman filter method.^51,59^

### 2.3 Reference DFT Calculations

DFT reference data were obtained using the RPBE functional applying the D3 dispersion correction.^61,62^ All DFT calculations were carried out using the mixed Gaussian orbital/plane wave package CP2K.^63,64^ A very high plane wave cutoff of 2500 Ry and a relative cutoff of 160 Ry were employed unless stated otherwise.^65^ Such a high cutoff is required for accurate forces and energies as has been previously shown.^48^ Gaussian orbitals were constructed using the triple-ζ TZV2P basis set, which includes polarization functions, for all atoms but Cesium, where a TZV2P-MOLOPT basis set was used.^66^ Furthermore, we employed norm-conserving Goedecker–Teter–Hutter (GTH) pseudopotentials for all atoms, tailored to the RPBE functional.^67–71^ This DFT calculation protocol has been shown to yield accurate results for aqueous systems, as demonstrated in previous work.^16,72–76^ For calculations that include homogeneous electric fields, we used the Umari and Pasquarello approach, as implemented in the CP2K quickstep module.^64,77^

### 2.4 Reference Configurations from AIMD

To provide configurations for initial training of the c-NNP, we performed ab initio molecular dynamics (AIMD) simulations using the CP2K quickstep module.^63,64^ The same functional, basis sets, and pseudopotentials were used as described in the Reference DFT Calculation section. For AIMD, the plane wave cutoff employed was reduced to 600 Ry and the relative cutoff was 20 Ry because the purpose of AIMD is merely to generate reasonable reference configurations for the c-NNP training (accurate reference forces and energies are then recalculated on the chosen training configurations using the higher cutoffs stated above). The simulation cells were constructed as follows: a structure was taken from a previous AIMD simulation of Na^+^ solvated in 64 waters.^76^ This structure’s original simulation cell was generated, via a similar protocol as outlined below (water box creation at fixed density, ion addition, and cell length adjustment according to partial molar volumes),^78^ starting with a water density of 0.997 kg L^–1^.^21^ Simulation cells were generated for aqueous Li^+^ and Cs^+^ by replacing Na^+^ with Li^+^ or Cs^+^, respectively, and adjusting the volume of the simulation cell by the difference in partial molar volumes between the ions and Na^+^.^78^ The final cell lengths for aqueous Li^+^, Na^+^, and Cs^+^ were 12.407, 12.406, and 12.486 Å, respectively. AIMD was run for 20 ps in the *NVT* ensemble for aqueous Li^+^ and Na^+^ and for 80 ps for Cs^+^. The time step was 0.5 fs, and massive Nosé–Hoover chains were employed with a 167 fs time constant to maintain the temperature at 300 K.

### 2.5 Generation of Simulation Cells for c-NNP MD

In order to generate unit cells for single cation solutions across a range of simulation cell sizes, the following procedure was used: first, we generated cubic unit cells of water molecules at a density of 0.99659 kg L^–1^ containing *N* water molecules, *N* = 64,128,256,512,1024. This is achieved by randomly placing water molecules into the cubic box and checking for overlaps until all waters were placed. Each of these unit cells were then equilibrated using the SPC/E water model for 100 ps in the *NVT* ensemble at 300 K with a 0.5 fs time step, with CP2K.^63,79^ The thermostat applied to maintain the system at 300 K was a Nosé–Hoover chain with a chain length of 3 and a time constant of 100 fs.^57,58^ Upon generating equilibrated water simulation cells, single cations were then added into the middle of the box. The volume of the simulation cell was then adjusted using partial molar volumes.^78^ Then, these single cation simulation cells were equilibrated for 100 ps in the *NVT* ensemble at 300 K with a 0.5 fs time step, with CP2K and an SPC/E alkali ion model.^17,63^ The same thermostat was applied as described above.^57,58^ The final dimensions of the simulation boxes are summarized in Table S4.

### 2.6 c-NNP MD (Zero-Field) Simulations

For the calculation of structural properties (radial and angular distribution functions), potentials of mean force and lifetimes of water ligand binding, 500 ps of PNNP MD were performed with the field strength set to 0, using the protocol outlined in Section 2.8. In order to calculate the diffusion constant for each ion at each concentration, the procedure outlined in ref 80 was used. First, positions and velocities were sampled from a 2 ns NVT trajectory every 6.25 ps. They were taken as initial conditions for 320 20 ps long c-NNP MD simulation runs in the *NVE* ensemble with a 0.5 fs time step for each system. For each of the 320 trajectories, the velocity autocorrelation function (VACF) was calculated,

6 $$VACF\left(\tau \right)=\left\langle v\left(t_{0}\right)v\left(t_{0}+\tau \right)\right\rangle$$

and the diffusion constant, *D*, obtained using the Green–Kubo relation,

7 $$D=\frac{1}{3}\int_{0}^{\infty }VACF\left(\tau \right)d\tau$$

In practice, we integrated the VACF only over 2.5 ps after which the integral was converged. From the 320 estimates of diffusion constants for each system, we drew 10,000 samples of size 320 with replacement and calculated the average diffusion constant for each resample yielding a distribution of diffusion constants for each cation and concentration. The mean of this distribution was taken as the final estimate of the diffusion constant, with the standard deviation being the error. Finally, plotting the diffusion constants vs 1/*L*, where *L* is the length of the simulation box, we performed a weighted linear fit (*y* = *mx* + *c* model) with an intercept of *D*_∞_, the infinite dilution diffusion constant. Finally, to calculate the molar ionic conductivity from the infinite dilution diffusion constant, *D*_∞_, we used the Nernst–Einstein relation:

8 $$\Lambda_{m}=\frac{F^{2}D_{\infty }}{RT}$$

where *F* is Faraday’s constant, *R* is the ideal gas constant, and *T* is the temperature of the system, 300 K.

### 2.7 APTNN Training

The APT can be efficiently calculated using the (exact) identity,

9 $${\frac{\partial M_{\zeta }}{\partial r_{i,\eta }}|}_{\varepsilon =0}={\frac{\partial F_{i,\eta }}{\partial \varepsilon_{\zeta }}|}_{\varepsilon =0}$$

where *F*_*i*,η_ is the force along the Cartesian coordinate η. Once the c-NNP was able to successfully simulate 1 ns of simulation length for all systems, 100 configurations were randomly extracted from the 128 water molecule simulation cells for each cation. APTs were obtained according to eq 9 by DFT finite difference calculations using the same electronic structure protocol as described in the Reference DFT Calculation section above. The electric field increment used for the finite difference calculation was 0.0257 V Å^–1^. APTNN learning was done using the E(3)-equivariant graph neural network e3nn^81–85^ that is based on the PyTorch library.^86^ The 100 configurations were split into batches between 20 and 80 configurations for training, and 20 configurations were retained for testing the APTNN. The settings for the APTNN were as follows: 8 committee members were trained, spherical harmonics up to *l* = 2 were used for the steerable basis, a radial cutoff of 6 Å was used, and 2 message passing layers were employed.

In exact theory, the APTs of the atoms obey the acoustic sum rule:^87–89^

10 $$\sum_{i}^{atoms}\frac{\partial M_{\zeta }}{\partial r_{i,\eta }}=q_{tot}\delta_{\zeta \eta }$$

where *q*_tot_ is the total charge of the system. This constraint is not imposed in the APTNN training. Instead, any deviation from the sum rule is corrected by evenly distributing the error across all of the atoms in the system. This ensures that the total charge, *q*_tot_, remains conserved when the field-induced forces, which are related to the APT according to eq 5, are applied.^43,44,90^ This scheme was successfully employed in previous work and shown to yield accurate results for liquid water.^49^

### 2.8 PNNP MD (Finite-Field) Simulations

The PNNP MD simulations were carried out using the APTNN and CP2K codebases, linking the two by using the i-Pi socket interface.^49,63,91^ Finite field simulations of the aqueous cations were carried out as follows. First, we took a configuration from a c-NNP (i.e., zero field) MD simulation of a cation in 128 water molecules. This was used as the starting configuration for a 50 ps PNNP MD simulation with a field of 2.57 × 10^–3^ V Å^–1^ applied along the *z*-direction using a time step of 1 fs and a CSVR thermostat with a time constant of 300 fs and a massive region.^92^ The CSVR thermostat was used to ensure that the system remained at 300 K, and the massive region ensured that the system maintains liquid-like properties.^93^ The field was then sequentially stepped up in increments of 2.57 × 10^–3^ V Å^–1^ from 2.57 × 10^–3^ V Å^–1^ to 2.57 × 10^–2^ V Å^–1^, followed by stepping in increments of 5.14 × 10^–3^ V Å^–1^ from 2.57 × 10^–2^ V Å^–1^ to 5.14 × 10^–2^ V Å^–1^. Finally, the field was stepped from 5.14 × 10^–2^ V Å^–1^ to 2.06 × 10^–1^ V Å^–1^, in increments of 2.57 × 10^–2^ V Å^–1^. The final configuration and velocity at 50 ps of the previous field was used as the starting configuration for the next field. The simulations for all field strengths were then extended to 200 ps. From these 200 ps simulations, 100 configurations were randomly sampled for each field and used as a test set for evaluation of the accuracy of PNNP against DFT. These trajectories were also used to calculate the ionic conductivity via linear regression of the current density against the applied field strength according to eq 16. The ionic current density was only regressed along the *z*-axis, in the linear regime, up to field strengths of 0.0514 V Å^–1^, excluding the first 20 ps as equilibration. For the calculation of structural properties (radial and angular distribution functions), potentials of mean force, and lifetimes of water ligand binding, we extended the 200 ps PNNP MD trajectories to 500 ps for a subset of fields (0.026, 0.051, 0.103, and 0.206 V Å^–1^). The first 20 ps were again excluded as equilibration for those analyses.

### 2.9 Vehicular and Structural Contributions to Ionic Current Density

To calculate the current density breakdown figures, we use the extended 480,000 frame PNNP MD simulations at a 1 fs time step. Along this trajectory, we calculated the integer coordination number using the radial cutoff distances displayed in Figure 1. Next, we calculate the velocity density of states (VDOS) for the ion at zero-field from the 480,000 frame trajectories, by taking the Fourier transform of the VACF. From the VDOS, we identify the lowest maximum intensity frequency, which corresponds to the rattling motion of the ion in the solvent cage. In order to calculate smooth VDOS plots to identify peaks, the trajectory was split into 24, 48, 96, 192, and 384 blocks of lengths 20,000, 10,000, 5000, 2500, and 1250 frames, respectively. The VACF and VDOS were calculated for each block and then averaged to yield a smooth VDOS. A comparison of the VDOS across the number of blocks from which it was computed was performed to ensure that a peak as chosen was not sensitive to the number of blocks. From this frequency, we calculate the rattling lifetime, which is used as a minimum lifetime for an *n*-fold coordinated ion to exist for, to be considered a stable species. The justification for this is that for a bond to be considered stable, it must exist for longer than the time period of its vibrational motion.^72^ In the case of an aqueous ion complex, the rattling motion is the vibrational motion of the ion in its solvation shell, and thus, the rattling lifetime is the minimum lifetime for an *n*-fold coordinated ion to be considered stable. All other configurations are considered labile and thus are counted in the “structural” current density contribution bin. This in turn allowed us to break the current density time series down into contributions from *n*-fold coordinated ions that exist for longer than their rattling lifetime, and those that are labile, i.e., exist for less than their rattling lifetime. The wavenumbers and lifetimes of the rattling motion are summarized in Table S5.

## 3 Results

In the PNNP MD approach, the interaction of the atomistic system with a homogeneous external electric field is treated perturbatively as discussed in ref 49. Following that logic, we train two ML models for each aqueous ion, a standard c-NNP for the unperturbed (zero-field) energy and forces and one for the APT. The latter is used to calculate the force perturbation due to the interaction with the external field. We use a committee^51^ of second-generation c-NNPs to model the unperturbed potential energy and forces and an E(3)-equivariant graph neural network to model the APT (APTNN). Details of the PNNP method and of the learning procedure are given in Methods and in ref 49.

All results presented below were obtained from PNNP MD simulations of a single cation (Li^+^, Na^+^, or Cs^+^) in 128 water molecules at 300 K, corresponding to a nominal cation concentration of 0.43 M in our periodic simulation cell. When an electric field is applied to the aqueous ion solutions, the ion will start to move along the field direction, and after some time, the system will reach a steady state where the velocity of the ion remains roughly constant. The work done on the system by the field is converted into excess thermal energy that we remove from the system using a stochastic thermostat with a target temperature of 300 K, which has been shown to be necessary to maintain fluid behavior in finite-field nonequilibrium simulations.^93^ All finite-field properties presented below were averaged over molecular dynamics trajectories simulated with PNNP MD in the steady state (see the Methods section for further details).

We begin by assessing the accuracy of the ML forces against DFT calculations for configurations taken from PNNP MD simulations at different field strengths. The results are summarized in Figures S1 and S2. The root-mean-square error (RMSE) in the total force and in its unperturbed force contribution is reasonably small, about 35 meV Å^–1^ for each ion, while the error in the perturbed force contribution is about 1–2 orders of magnitude smaller, in line with the smaller magnitude of the perturbed forces compared to the unperturbed forces. Unsurprisingly, the error for the perturbed force increases with field strength because the APT error is magnified by the field strength. A commonly used force error metric,^56^ the ratio between force RMSE and force root-mean-square-fluctuations relating the actual RMSE to the intrinsic fluctuations of the property along the MD trajectory, is also reasonably small—between 6 and 10% for perturbed and unperturbed force contributions. Finally, we report the atomic polar tensor species RMSE and parity plots in Figure S3. Overall, all error metrics are even slightly better than in our previous investigation of pure liquid water.^49^

The ion-oxygen radial distribution functions of the ions at zero field, as obtained from c-NNP MD simulations, are shown in Figure 1. They are in good agreement with the results from many-body potential-based molecular dynamics simulations,^19^ which we consider as a benchmark for our simulations (see Figure S4 for a comparison). Li^+^ is predominantly tetrahedrally coordinated, though 3-fold and 5-fold coordination is also observed on the nanosecond (ns) time scale of current simulations. Na^+^ is predominantly 6-fold coordinated with a significant population of 5-fold coordinated structures and a small fraction of 7-fold coordination. In contrast to Li^+^ and Na^+^, Cs^+^ does not form a stable first shell coordination sphere. We observe very frequent water exchange events (lifetimes are discussed further below), giving rise to a broad distribution of coordination numbers that average to about 10 water molecules.

**Figure 1:**
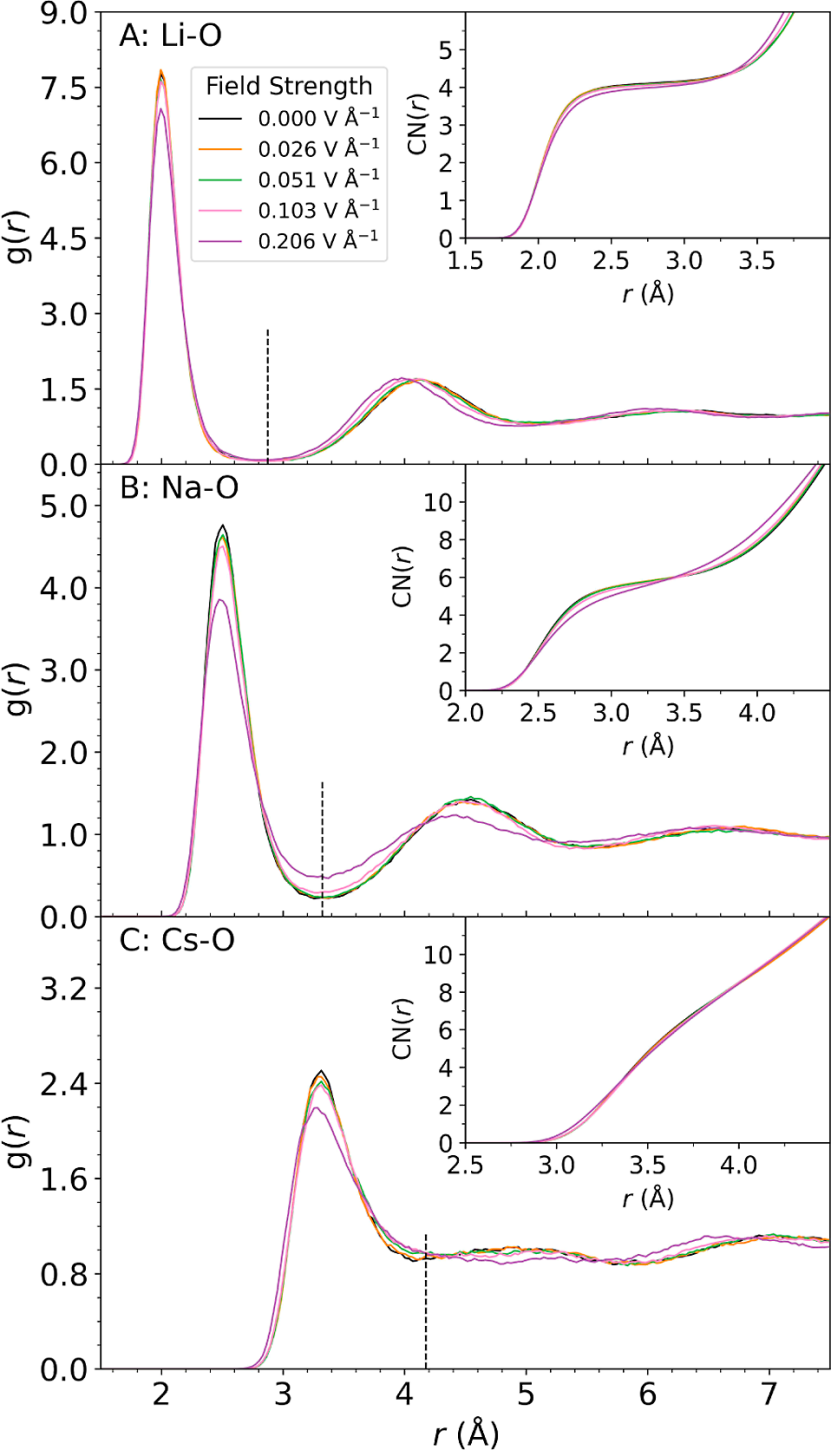
Ion-oxygen radial distribution functions (RDF) for aqueous alkali ions at different external electric field strengths. The RDFs shown for Li_(aq)_^+^ (A), Na_(aq)_^+^ (B), and Cs_(aq)_^+^ (C) were averaged over PNNP MD trajectories of length 480 ps and collected in bins of width 0.03 Å. The insets display the ion-oxygen coordination number obtained by integration of the RDF. The radius of the first solvation shell at zero-field, corresponding to the first minimum of the RDF, is indicated by vertical dashed lines. Notice the flattening of the minimum between first and second solvation shell from Li_(aq)_^+^ to Cs_(aq)_^+^ reflecting the weakening of the ion–water interactions.

We find that the radial distribution functions remain rather insensitive to the presence of electric fields. Even at the highest field strengths applied, 0.206 V Å^–1^, changes are rather subtle (see Figure 1 and Table S1). Yet, the electric field does have a strong effect on ion solvation, as is best illustrated by plotting the potential of mean force (PMF) along the coordination number (CN), Figure 2, calculated according to:

11 $$PMF\left(CN\right)=-k_{B}T\;\ln\left[p\left(CN\right)\right]+const$$

12 $$CN=\sum_{j}^{N_{O}}\frac{1-{\left(\frac{r_{j}}{R_{0}}\right)}^{NN}}{1-{\left(\frac{r_{j}}{R_{0}}\right)}^{ND}}$$

where *p*(CN) is the probability to observe a given value of the coordination number along a MD trajectory, *k*_B_ is the Boltzmann constant, *T* the temperature, *r*_*j*_ is the distance between the ion and oxygen atom *j*, *R*_0_ is the position of the first minimum of the ion-oxygen radial distribution function, *N*_O_ is the number of oxygen atoms, and NN = 20 and ND = 40 are constants. We observe significant changes to the PMF at large field strengths (0.1–0.2 V Å^–1^), in particular for Na^+^: the minimum at CN = 6 flattens considerably in both directions leading to an increase in population of 5-fold and 7-fold coordination structures and thus to a less well-defined coordination shell. For Li^+^, the PMF with a minimum at CN = 4 flattens only in the direction toward smaller coordination numbers, resulting in an increase in the fraction of 3-fold coordination. By contrast, the PMF for Cs^+^ appears to be very robust, even at the highest field strengths applied. The CN distribution increases only very slightly, and the mean value of CN remains unchanged. Overall, the effect of strong electric fields on Li^+^ and Na^+^ is a flattening of the PMF and an increase in the population of coordination species that are a minority species at zero field, while Cs^+^ remains largely unperturbed. Using the first solvation shell radial cutoff at zero field, displayed by a dashed black line in Figure 1 and reported in Table S1, we also report histograms of the integer coordination numbers at different field strengths in Figure S5. The same trends as discussed above occur for the integer coordination numbers.

**Figure 2:**
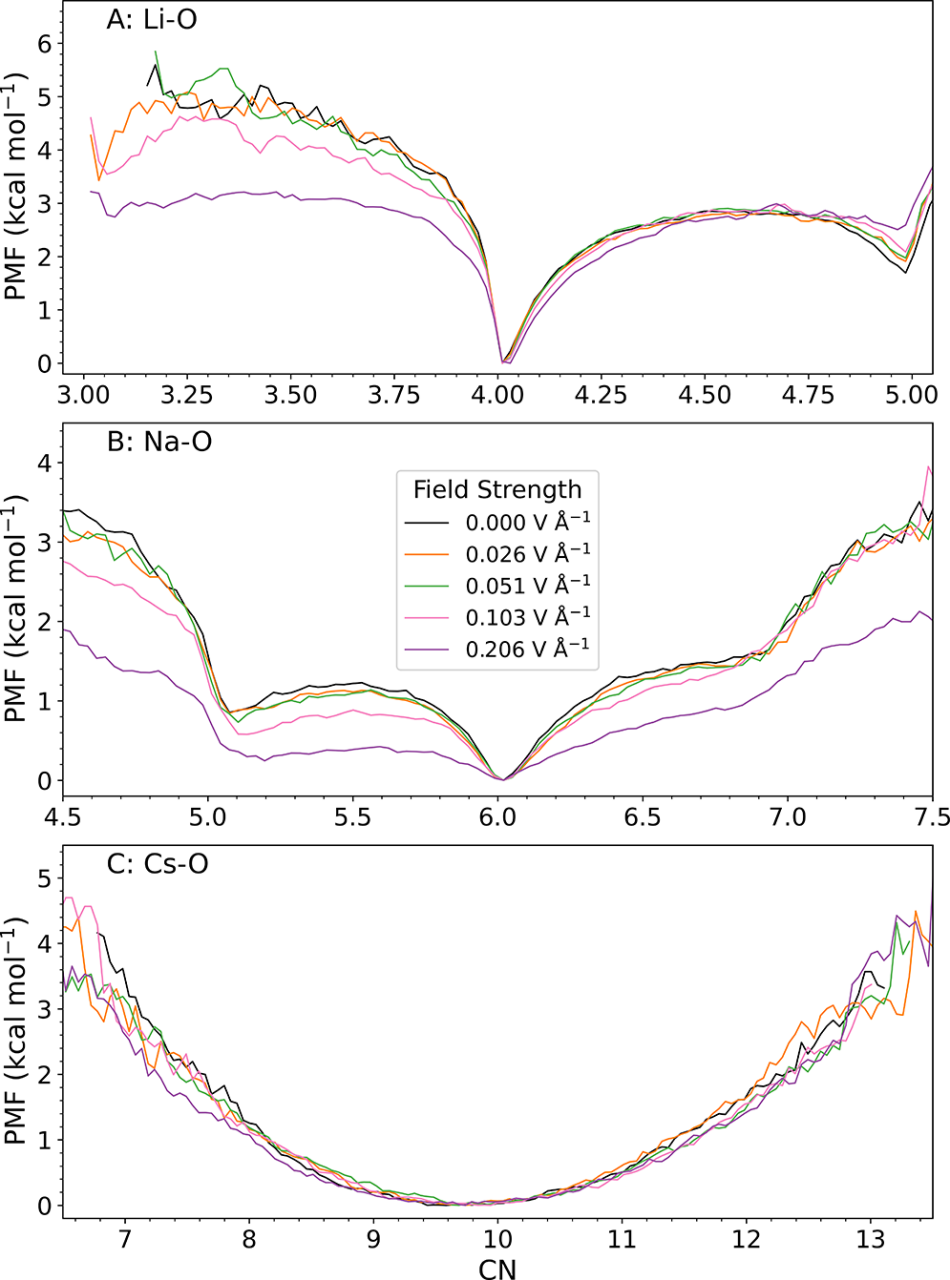
Potential of mean force (PMF) as a function of the ion-oxygen coordination number (CN) for different field strengths. PNNP MD trajectories of length 480 ps are used to calculate the PMF (eq 11) along the coordination number (eq 12) for Li_(aq)_^+^ (A), Na_(aq)_^+^ (B), and Cs_(aq)_^+^ (C). Notice the marked flattening of the PMF for Na_(aq)_^+^ at high fields and the near insensitivity of the PMF for Cs_(aq)_^+^ upon application of external fields.

To rationalize the strong electric field effect on ion coordination, we define two angles, the angle α between the vector pointing from a first shell oxygen atom to the ion and the vector pointing in the field direction, and the tilt angle of the first shell water molecules, θ (see Figure 3 for a schematic). The joint probability density for cos­(α) and cos­(θ) averaged over all first shell water molecules and simulation time is shown in Figure 3. Note that in Figure 3, we plot the joint distribution of cos­(α) and cos­(θ), conditional on the water molecule being in the first solvation shell of the ion. This ensures that we avoid solid angle weighting effects that would arise if we plotted the joint distribution of α and θ directly. The angle α indicates whether the water ligand is leading (cos­(α) < 0, α > 90°) or trailing the cation (cos­(α) > 0, α < 90°) with respect to the direction of the applied electric field. In the former case, the water dipole is favorably aligned and in the latter case unfavorably aligned with respect to the applied electric field. At zero field (panels A–C), there is no correlation between cos­(α) and cos­(θ), as expected for an isotropic system. We observe cos­(α) to be uniformly distributed between −1 and 1 due to the random rotational orientation of the first shell coordination complex at zero field. The mean values for cos­(θ) are 0.73, 0.63, and 0.43 (corresponding to tilt angles of 43.0°, 51.3°, and 64.6°) for Li^+^, Na^+^, and Cs^+^, respectively. Furthermore, the spread of the cos­(θ) distribution increases in the order Li^+^ < Na^+^ < Cs^+^. These trends in mean and spread are in line with the charge density of the ions: the smaller the cation, the higher the charge density and the smaller the tilt angle to minimize repulsive cation–water dipole interactions.

**Figure 3:**
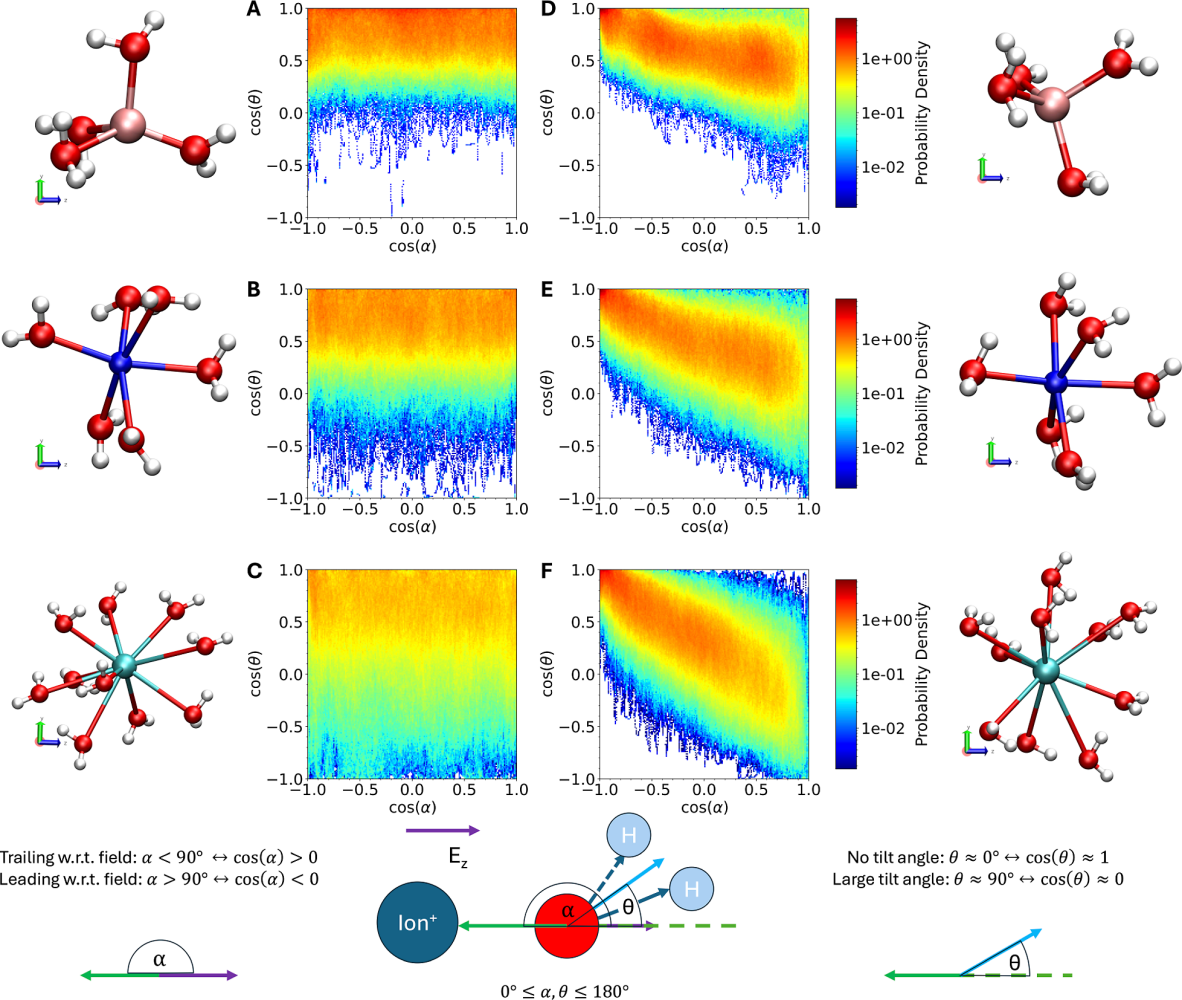
Field-induced changes in the tilt angle of the first solvation shell water molecules. The plots show the probability density of cos­(θ) and cos­(α), where θ is the tilt angle of the water dipole with respect to the field direction and α is the angle between the vector pointing from a first shell oxygen atom to the ion and the external electric field vector. When cos­(α) < 0 or α > 90° (cos­(α) > 0 or α < 90°), the water molecule is leading (trailing) the ion relative to the applied external electric field, see schematic at the bottom of the figure. The data presented are collected from 480 ps of PNNP MD for Li_(aq)_^+^, Na_(aq)_^+^, and Cs_(aq)_^+^ at zero field (panels (A–C)) and at a field strength of 0.206 V Å^–1^ (panels (D–F)). The probability density is zero in regions of white and increases from blue to red, as shown in the probability density color bar, which is on a logarithmic scale. Alongside each plot is a representative snapshot of the first solvation shell taken from a PNNP MD simulation of the aqueous ions, with the ion at the center, oxygen atoms depicted in red spheres, and hydrogen atoms in white spheres and the electric field pointing from left to right (higher solvation shells have been removed for clarity). No correlation is observed between cos­(θ) and cos­(α) at zero field, whereas a strong correlation is observed at finite electric field: first shell water molecules ahead of the ion relative to the applied field (cos­(α) < 0, α > 90°) exhibit decreased tilt angles, corresponding to enhanced alignment with the external field, whereas trailing water molecules (cos­(α) > 0, α < 90°) exhibit increased tilt angles. This highlights the competition between ion–water and water–field interactions.

When the field is present (panels D–F), cos­(α) and cos­(θ) become correlated: water molecules leading the ion (cos­(α) < 0, α > 90°) have cos­(θ) values of 0.70, 0.63, and 0.54 (corresponding to tilt angles of 45.8°, 51.0°, and 57.1°) for Li^+^, Na^+^, and Cs^+^, respectively. These values are similar to the average over all first shell water molecules at zero field for Li^+^ and Na^+^ but slightly smaller for Cs^+^. This is likely due to the increased radius of the solvation shell of Cs^+^ allowing for greater orientational freedom of the water dipoles, relative to Li^+^ and Na^+^. Conversely, the water molecules trailing the ion (cos­(α) > 0, α < 90°) now adopt significantly smaller cos­(θ) values of 0.49, 0.30, and 0.05 (corresponding to tilt angles of 60.7°, 72.5°, and 87.2°) for Li^+^, Na^+^, and Cs^+^, respectively. This latter response represents a trade-off: the increase in the tilt angle decreases the unfavorable interactions of the trailing water dipoles with the field; however, it also increases the unfavorable interaction of the trailing water dipoles with the cation. The strength of the applied field, along with the charge density and size of the ion, determines the balance between these two competing effects. The correlation (slope) in panels (D-F) notably increases in the order Li^+^ < Na^+^ < Cs^+^ consistent with the decreasing strength/increasing lability of the ion–water coordination bond and ionic size. Moreover, the area of the tilt angle distribution markedly decreases as the field constrains the thermal fluctuations of the water dipoles relative to the fluctuations at zero field.

These considerations help us understand the electric field effect on the PMF, presented above and in Figure 2. The dipole moment of the trailing water molecules, despite the increase in their tilt angle, is still energetically unfavorably aligned with respect to the field. As a result, the barrier for their dissociation from the ion is decreased, explaining the decrease in the PMF toward lower coordination numbers (panels (A) and (B)). On the contrary, one might expect that the barrier for addition of a *leading* water molecule with its dipole moment aligned parallel to the field becomes more favorable when the field is present. This would explain the decrease in PMF in the direction of increasing coordination number for Na^+^ (panel (B)). A similar decrease in PMF is absent for Li^+^ (panel (A)) likely because the ion is too small to accommodate an additional water ligand. Interestingly, although the asymmetry in the tilt angles of trailing and leading water molecules is the largest for Cs^+^, it results in the smallest effect on the PMF and mean coordination number. This is likely because the radius of the first coordination sphere of Cs^+^ is so large (4.2 Å) and the charge of the ion so diffuse, that a misaligned water molecule in the first solvation shell (hydrogens pointing toward the ion) does not incur a major energetic penalty.

Next, we investigate the effect of the external field on the continuous lifetime of first shell water ligands, τ_c_, defined by:^94^

13 $$\tau_{c}=\int_{0}^{T}d\tau C\left(\tau \right)$$

14 $$C\left(\tau \right)={\left\langle \frac{S\left(t_{0},t_{0}+\tau \right)}{N\left(t_{0}\right)}\right\rangle }_{t_{0}}$$

where *C* is the continuous survival correlation function (or weighted-origin survival probability) and *T* the length of the trajectory. In eq 14, *N*(*t*_0_) counts the number of first shell ligands within the distance *R*_0_ at time *t*_0_ and *S*(*t*_0_, *t*_0_ + τ) counts how many of those same water molecules remain continuously within *R*_0_ until time *t*_0_ + τ. The brackets ${\left\langle \cdot \cdot \cdot \right\rangle }_{t_{0}}$ denotes averaging over all initial times *t*_0_.

The continuous survival correlation function and the lifetimes are shown in Figure 4, and the latter are summarized in Table S2. At zero field, the solvation shell of Li^+^ exhibits the longest lifetime (20 ps) followed by Na^+^ (6 ps) and Cs^+^ (2 ps). This trend is consistent with the decreasing free energy barrier from Li^+^ > Na^+^ > Cs^+^ for a change in coordination number, Figure 2. While not directly comparable to the residence lifetimes calculated from many-body potential-based MD in ref 19 (as they employ intermittent correlation functions), the trends observed are qualitatively similar. With increasing field strengths, the lifetimes tend to decrease, to 14 ps for Li^+^ and to 3 ps for Na^+^ at 0.2 V Å^–1^. Yet, the lifetime of the solvation shell for Cs^+^ is virtually unaffected by the presence of an external field. These results are again consistent with the field-dependence of the PMFs.

**Figure 4:**
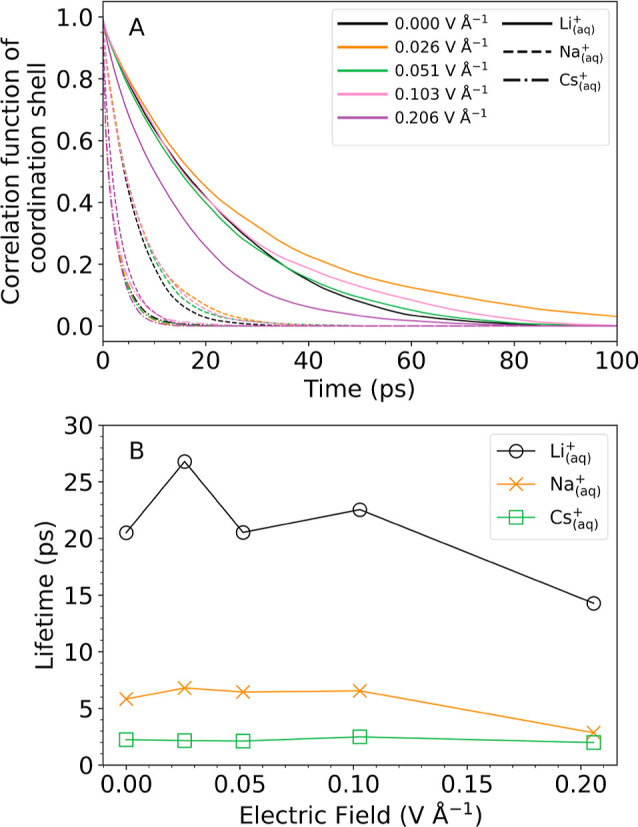
Lifetimes of first shell water molecules for different field strengths. Continuous correlation functions are calculated according to eq 14 (panel A) and integrated according to eq 13 to obtain the continuous lifetimes (panel (B)). The correlation functions are obtained from PNNP MD simulations for Li_(aq)_^+^ (solid lines), Na_(aq)_^+^ (dashed lines), and Cs_(aq)_^+^ (dash dotted lines) at different field strengths as indicated. Numerical values of the lifetimes are summarized in Table S2.

We now present the major result of this work, the ionic conductivity obtained from finite-field PNNP MD simulations. Applying a field with magnitude *E*_*z*_ along the *z*-direction, the instantaneous ionic current density along *z* due to ion migration, *J*_ion,*z*_, can be conveniently obtained by

15 $$J_{ion,z}=\frac{1}{V}\sum_{\eta }\frac{\partial M_{z}}{\partial r_{ion,\eta }}\nu_{ion,\;\eta }=\frac{1}{V}\sum_{\eta }P_{ion,\eta ,z}\nu_{ion,\eta }$$

where $\frac{\partial M_{z}}{\partial r_{ion,\eta }}$ are elements of the APT for the ion, $P_{\mathrm{ion},\eta ,z}$ , which are already available from the PNNP simulations, and *v*_ion,η_ is the velocity of the ion along the Cartesian direction η and *V* is the volume of the simulation cell. To obtain the ionic conductivity, σ_ion_, the instantaneous current density eq 15 is averaged over PNNP MD runs at different field strengths giving ⟨*J*_ion,*z*_⟩(*E*_*z*_). At small fields, the increase in ⟨*J*_ion,*z*_⟩ with *E*_*z*_ is expected to be linear (i.e., linear response) and the ionic conductivity, σ_ion_, is defined as the slope in the linear regime,

16 $$\left\langle J_{ion,z}\right\rangle =\sigma_{ion}E_{z}$$

To convert to molar ionic conductivity, Λ_*m*_, we divide by the concentration of the cation in the simulation box, *c*_ion_, Λ_*m*_ = σ_ion_/*c*_ion_. The nominal concentration of the cation in our periodic simulations is 0.43 M, as previously stated for all of the results presented.

The ionic current densities versus electric field strengths are shown in Figure 5. For each ion, we observe a linear increase in ionic current density up to field strengths of about 0.05 V Å^–1^ followed by a sublinear increase for higher field strengths. The linear regime thus extends to larger field strengths than, e.g., the dipole polarization of pure liquid water (up to 0.0154 V Å^–1^ ^49^). However, the statistical errors in the current densities do not permit an accurate estimation of the upper limit of the linear response regime. A regression in the linear regime gives molar ionic conductivities of 45.7, 61.1, and 73.9 S cm^2^ mol^–1^ for Li^+^, Na^+^, and Cs^+^, in good agreement with the experimental values of 38.7, 50.1, and 76.8 S cm^2^ mol^–1^, respectively^96^ (numerical values are summarized in Table S3). Moreover, our computed values from finite-field PNNP MD simulations are in good agreement with results from zero-field c-NNP simulations using the Green–Kubo equation (48.9, 58.2, and 76.9 S cm^2^ mol^–1^, see Figure S6 and Methods).

**Figure 5:**
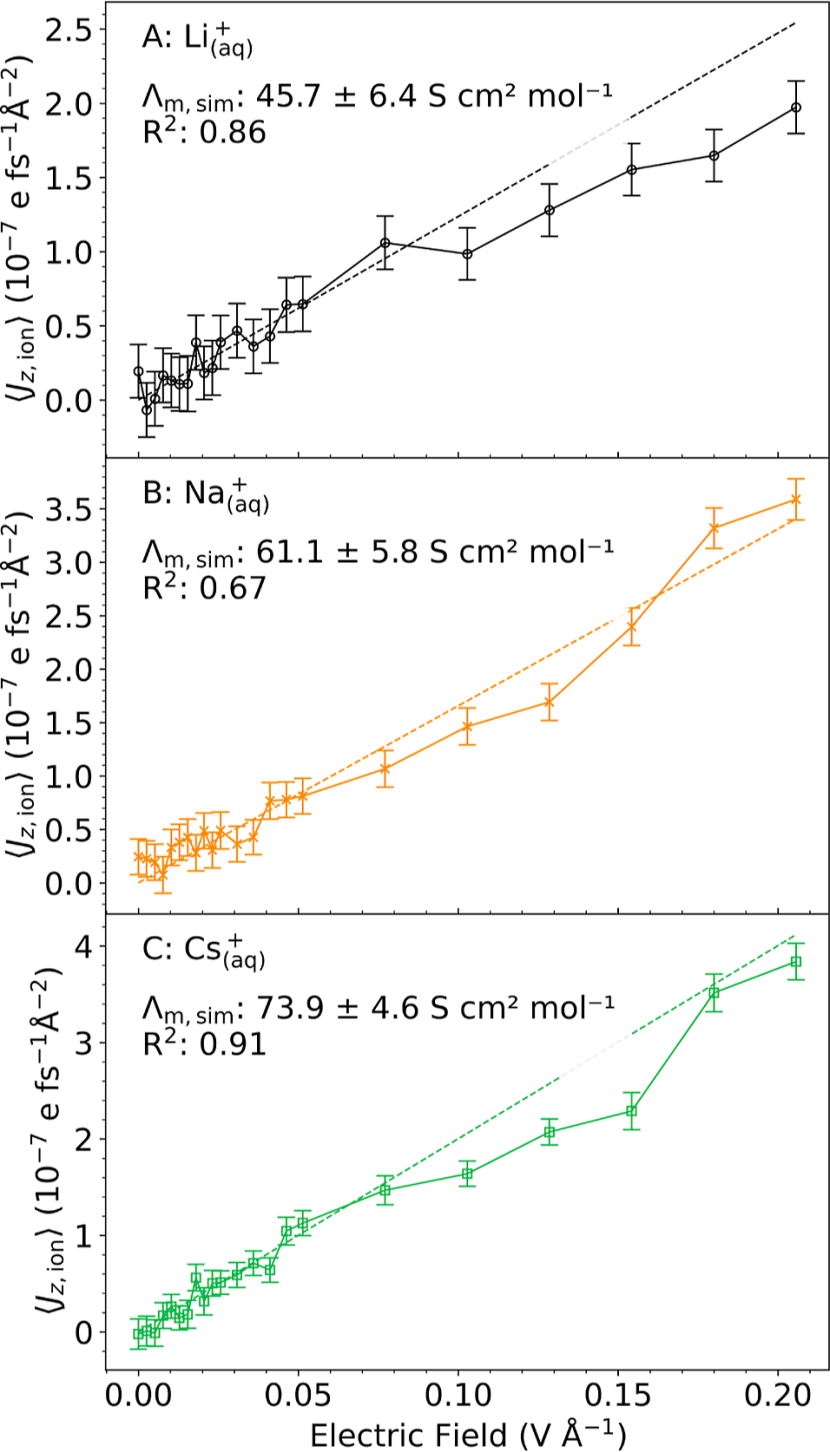
Ionic current density as a function of applied electric field. The instantaneous ionic current density, eq 15, is averaged over 180 ps PNNP MD trajectories run at different field strengths *E*_*z*_, to obtain the mean ionic current density, ⟨*J*_ion,*z*_⟩, for Li_(aq)_^+^ (A), Na_(aq)_^+^ (B), and Cs_(aq)_^+^ (C). The error bars indicated are the standard error of the mean calculated using a block averaging procedure to account for the statistical inefficiency of the time series.^95^ The linear regime was determined by visual inspection to extend up to 0.0514 V Å^–1^ for all ions. A linear fit was performed in this regime using a linear regression model with zero intercept and results in *R* ^2^ values as indicated. The ionic conductivity σ_ion_ is obtained as the slope of the linear fit according to eq 16 and converted to molecular ionic conductivities, Λ_*m*, sim_. Simulated and experimental molar ionic conductivities are summarized in Table S3.

We find that the conductivity of the heavy Cs^+^ ion is nearly 50% higher than that of the light Li^+^ ion, in agreement with the experiment. This is usually explained by the larger number of hydration shells that migrate together with the lighter ion as a consequence of its higher charge density compared to the heavier ion, as mentioned in the Introduction section. In the case of Li^+^ and Cs^+^, this picture is taken to the extreme resulting in qualitatively different migration mechanisms for the two ions: whereas Li^+^ migrates almost exclusively via a *vehicular* mechanism, Cs^+^ migrates exclusively via a *structural* mechanism at all field strengths (see below for a detailed analysis). In the vehicular mechanism, the ion migrates together with its first and possibly higher solvation shell(s) with the first solvation shell remaining intact, whereas in the structural mechanism, the ion hops between neighboring sites within the solvent without dragging any solvent molecules with it. Hence, in the vehicular regime, the identity of the first shell water molecules remains the same during migration, whereas in the structural regime, their identity keeps constantly changing.

In the following, the contribution of vehicular and structural migration to the current density, and hence ionic conductivity, is quantified by breaking down the PNNP trajectories into segments in which the ion is stably *n*-fold coordinated (*n* = 3–7, for Li^+^ and Na^+^). We consider the *n*-fold coordination as stable if it exists for at least one oscillation period of the rattling motion of the ion in the solvent cage (see the Methods section).^97,98^ A stable solvation shell is thus defined as when all coordinated waters remain within the first solvation shell cutoff for at least a single intermolecular ion–water cage vibration. All frames that do not belong to an *n*-fold coordinated segment are assigned as an unstable segment of the trajectory. The current density contribution for each stable *n*-fold coordinated species is then obtained by averaging the instantaneous current density, eq 15, over the frames in the corresponding *n*-fold coordinated segments. We denote these contributions as *n*-fold coordinated vehicular contributions to the current density (Figure 6, bars in color). The structural contribution to the current density is obtained by averaging the instantaneous current density in eq 15 over the frames in the unstable segments (Figure 6, bars in gray). The time-weighted sum of all *n*-fold coordinated vehicular contributions and structural contributions gives the total mean current density.

**Figure 6:**
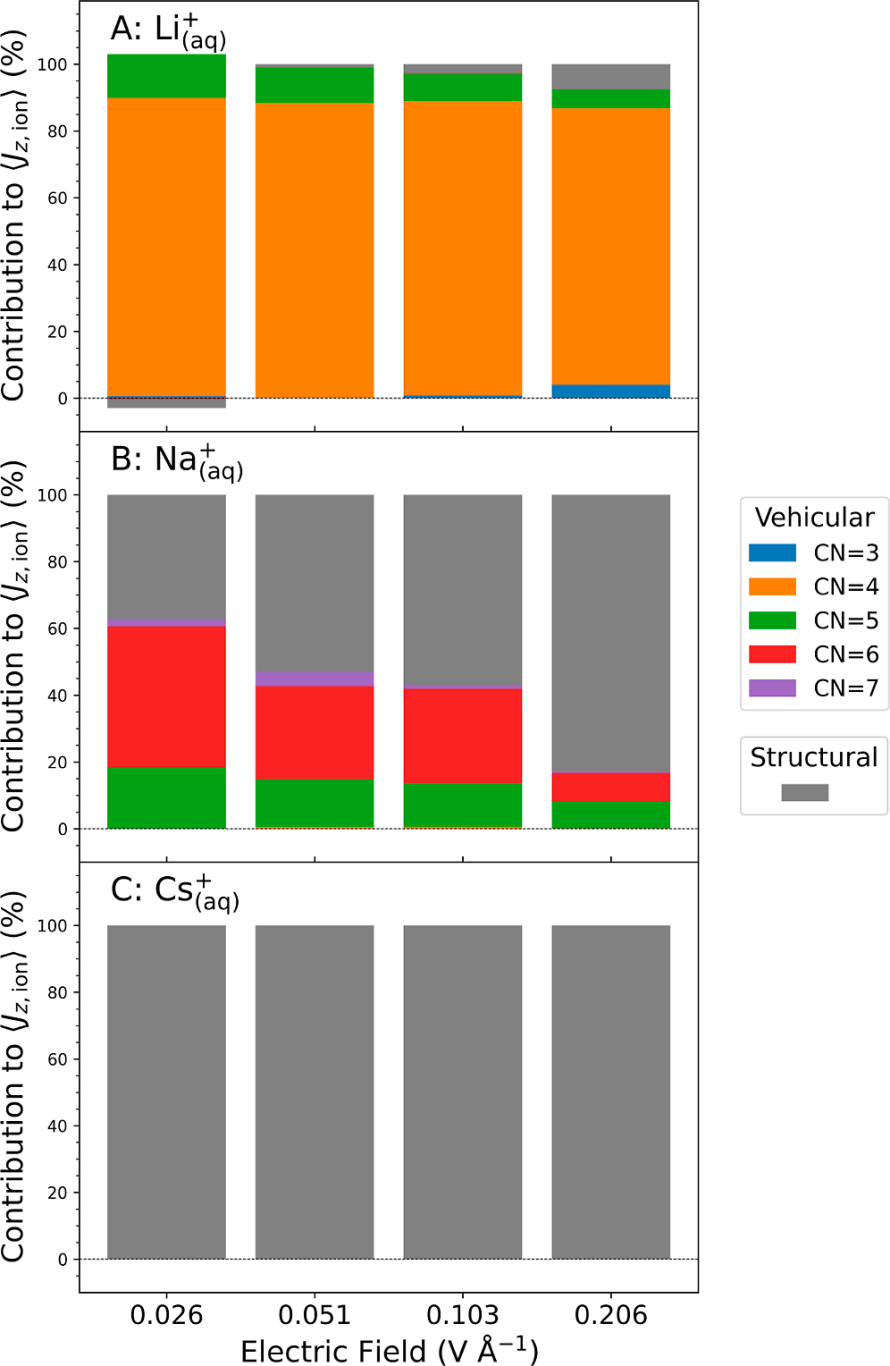
Breakdown of the ionic current density into contributions from vehicular and structural migration for Li_(aq)_^+^ (A), Na_(aq)_^+^ (B), and Cs_(aq)_^+^ (C). The bars in color indicate the vehicular contribution to the ionic current density from each stable *n*-fold coordinated species, while the bars in gray indicate the structural contribution to migration. For details of this analysis, we refer to the main text and the Methods section. Notice that Li_(aq)_^+^ migrates via a vehicular mechanism, whereas Cs_(aq)_^+^ migrates via a structural mechanism at all field strengths. For Na_(aq)_^+^, a transition from predominantly vehicular to predominantly structural diffusion is observed upon increase of the field strengths.

We find that for Li^+^, most of the ionic current density is due to vehicular migration of the 4-fold coordinated species as expected, with small contributions from 3-fold coordinated vehicular (<5%) and structural migration (<10%) appearing at the highest field strength at 0.2 V Å^–1^ (Figure 6A). By contrast, the current density for Cs^+^ is entirely due to structural migration at zero and any finite-field strengths (Figure 6C). Strikingly, in the case of Na^+^, we observe a transition from predominantly vehicular to predominantly structural migration as the electric field is increased. At low fields, about 2/3 of the current density are due to 6-fold and 5-fold coordinated vehicular migration of Na^+^ while 1/3 is due to structural migration (Figure 6B). At the highest field strength, the majority of current density (>80%) is due to structural migration. Interestingly, the transition from vehicular to structural migration is accompanied by a marked increase (revival) of the saturating current density outside the linear response regime (Figure 5B, with an increased slope between 0.13 and 0.18 V Å^–1^).

The prior decomposition of current density into vehicular and structural contributions is by no means unique and will always depend on some physically motivated choices. Here, *n*-fold vehicular transport is defined to occur when the coordination state persists longer than the time period for the rattling vibrational motion of the first solvation shell ion cage. This was determined consistently for each ion from the velocity autocorrelation function. This is a physically intuitive lower limit; if the coordination state is only transient and decays faster than the time period for the rattling vibration, then it must be classified as unstable. The rattling mode corresponds to the vibration of the ion within the *entire* first solvation shell cage, which is the relevant motion for ionic migration. It is sensitive to the mass of the ion and the strength of the ion–water interactions. Note that we employ both a physical stability criterion, all coordinated waters must be within the first solvation shell cutoff (approximating the potential of mean force barrier separating the first and second solvation shells), and a dynamical stability criterion, the time period for the rattling vibration of the ion in the solvent cage, to define stable coordination states. This time period ensures that the ion–water coordination bonds remain intact for a physically meaningful time period, specific to each ion. We emphasize that this decomposition is not based on arbitrary parameters but on physically motivated quantities that are explicitly calculated from the intrinsic dynamics of each ion in solution.

Alternatively, one could decompose the ionic current density by using the continuous or intermittent correlation function-based residence time or the *stable state* picture based on the potential of mean force.^99,100^ The continuous correlation function-based residence time is a trajectory averaged quantity based on persistent water identity, not instantaneous coordination state; thus, it is not suitable for decomposing ionic current density into vehicular and structural contributions. Furthermore, transient escapes of water molecules from the first solvation shell lead to a lower estimate of the residence lifetime, which would tend to skew our decomposition toward structural migration. The intermittent correlation function-based residence time requires the choice of a time threshold, t*, for which a given water molecule is allowed to leave and return to the first solvation shell without this failed exchange being considered as a change in the coordination state.^99^ Previous studies have shown that residence lifetimes via the intermittent correlation function are very sensitive to the choice of t*.^100^ Often in the literature, t* is chosen to be 2 ps in order to be consistent with the original definition by Impey et al. This is often applied ubiquitously regardless of ion species.^19,99^ The *stable state* picture based on the potential of mean force along the ion–water oxygen distance coordinate requires the definition of two sets of separatrices for reactant and product basins, which act as absorbing states.^100^ This approach in turn provides a more rigorous way to disregard transient ligand escapes and has been shown to provide more robust estimates of mean residence times.^100^ To the best of our knowledge, there is no canonical decomposition method for breaking down current density contributions into vehicular and structural components. In summary, we use a joint criterion based on a physically motivated dynamic time period and a structural stability measure. This provides a robust and intuitive way to decompose the ionic current density into vehicular and structural contributions, as all quantities used are explicitly calculated from the intrinsic dynamics of each ion in solution.

While alternative decompositions may change the quantitative contributions from vehicular and structural migration reported here, we expect the qualitative conclusions to remain robust. If the time period used to define stable coordination was increased (e.g., twice the time period for rattling vibration), then we would expect a reduced contribution from vehicular migration and an increased contribution from structural migration. For Cs^+^, which exhibits very labile coordination, increasing the time threshold would not change the conclusion that its migration is dominated by structural diffusion. For Li^+^, which exhibits very stable coordination, increasing the time threshold would not change the conclusion that its migration is dominated by vehicular diffusion, although the small contribution from the structural mechanism may increase slightly. Finally, for Na^+^, which exhibits intermediate coordination stability, increasing the time threshold may shift the balance further toward structural migration, earlier in the applied field strength range. Nonetheless, the key conclusion that Na^+^ exhibits a transition from predominantly vehicular to predominantly structural migration with increasing applied field strength would remain unchanged.

## 4 Conclusions

In this work, we have extended our recently introduced machine learning methodology to simulate electrified ionic solutions over multiple nanoseconds at AIMD quality. We found that the impact of external electric fields on the structure and dynamics of alkali cations does not simply scale with their charge density. Rather, there appears to be a favorable intermediate ion–water interaction energy regime, where important properties such as solvation structure, ligand residence times, and ion transport dynamics become strongly susceptible to the application of electric fields. Evidently, for Li^+^, the ion–water coordination bonds are too strong for its first solvation shell to be significantly perturbed by ambient electric fields, resulting in a vehicular ion transport mechanism at all field strengths investigated (<0.2 V Å^–1^). Conversely, for Cs^+^, the ion–water coordination bonds are so weak that the field-induced polarization of water molecules (even the ones close to Cs^+^) is hardly perturbed by the presence of the ion. As a consequence, Cs^+^ migrates via hopping between temporally unoccupied sites of polarized liquid water. Finally, Na^+^ emerges as a “Goldilocks” ion: the ion–water interactions are sufficiently strong to maintain a relatively stable solvation shell at zero field but sufficiently weak to be easily perturbed and frequently broken and replaced by other water molecules upon application of moderate to strong electric fields. As a consequence, both vehicular and structural transport regimes exist for Na^+^ depending on the strength of the electric field applied. Our results demonstrate that PNNP MD provides unprecedented atomistic insights into the interplay among solvation structure, dynamics, and ion transport under electric fields. Future investigations will focus on understanding electric field effects in concentrated ionic solutions and at solid–electrolyte interfaces.

## Supplementary Material



## Notes

Details on the calculation of water residence lifetimes, error metrices for forces and APTNN fitting, comparison of ion-oxygen radial distribution functions to many-body potential-based MD simulations, integer coordination number histograms, zero-field Green–Kubo ionic conductivity calculations, ion-oxygen radial distribution function information, water residence lifetimes, ionic-conductivity numerical values, simulation cell parameters, and ion rattling frequencies and time periods (PDF)
