# Supplementary material for: Transition from Vehicular to Structural Ionic Transport in Electrified Alkali Aqueous Solutions
Source: J Phys Chem B. 2026 Mar 2;130(10):2817–29. doi: 10.1021/acs.jpcb.5c07449 (PMC12990100; doi:10.1021/acs.jpcb.5c07449)
Supplement: Supplementary file 1 [file jp5c07449_si_001.pdf]

Supplementary Information: Transition from Vehicular to Structural Ionic Transport in Electrified Alkali Aqueous Solutions

Kit Joll,[†] Philipp Schienbein,[†,‡,¶] Kevin M. Rosso,[§] and Jochen Blumberger[*,†]

†*Department of Physics and Astronomy and Thomas Young Centre, University College London, London, WC1E 6BT, United Kingdom*

‡*Lehrstuhl für Theoretische Chemie II, Ruhr-Universität Bochum, 44780 Bochum, Germany*

¶*Research Center Chemical Sciences and Sustainability, Research Alliance Ruhr, 44780 Bochum, Germany*

§*Pacific Northwest National Laboratory, Richland, Washington 99354, United States*

E-mail: j.blumberger@ucl.ac.uk

Calculation of lifetimes of water ligands

We follow the work of Rapaport et al. and define a state variable $s_i(t)$:[1]

$$s_i(t) = \begin{cases} 1, & \text{if molecule } i \text{ is continuously in the first solvation shell between } t_0 \text{ and t} \\ 0, & \text{otherwise.} \end{cases}$$

This can be expressed in terms of products of indicator functions like:

$$s_i(t) = \prod_{t'=t_0}^{t} \mathbb{1}_i(t')$$

where $\mathbb{1}_i(t')$ is 1 if molecule i is in the first solvation shell at time t' and 0 otherwise. Rapaport then defines the continuous correlation function for a given time origin, t_0, as:

$$C(\tau|t_0) = \frac{\sum_i s_i(t_0)s_i(t_0+\tau)}{\sum_i s_i(t_0)} = \frac{\sum_i \mathbb{1}_i(t_0)\prod_{t'=t_0}^{t_0+\tau} \mathbb{1}_i(t')}{\sum_i \mathbb{1}_i(t_0)} = \frac{\sum_i \prod_{t'=t_0}^{t_0+\tau} \mathbb{1}_i(t')}{\sum_i \mathbb{1}_i(t_0)}.$$

Averaging over all valid time origins t_0 for a given τ then yields the continuous correlation function:

$$C(\tau) = \frac{\sum_{t_0=0}^{T-1-\tau} \sum_i \prod_{t'=0}^{\tau} \mathbb{1}_i(t_0+t')}{\sum_{t_0=0}^{T-1} \sum_i \mathbb{1}_i(t_0)},$$

where T is the total trajectory length. By defining $S(t_0, t_0+\tau) = \sum_i \prod_{t'=0}^{t_0+\tau} \mathbb{1}_i(t_0+t')$ as the number of water molecules that remain continuously in the first solvation shell from time t_0 to $t_0+\tau$ and $N(t_0) = \sum_i \mathbb{1}_i(t_0)$ as the number of water molecules in the first solvation shell at time t_0, we can rewrite the continuous correlation function as:

$$C(\tau) = \frac{\sum_{t_0=0}^{T-1-\tau} S(t_0, t_0+\tau)}{\sum_{t_0=0}^{T-1} N(t_0)} = \sum_{t_0=0}^{T-1-\tau} \frac{N(t_0)}{\sum_{t_0'=0}^{T-1} N(t_0')} \frac{S(t_0, t_0+\tau)}{N(t_0)} = \left\langle \frac{S(t_0, t_0+\tau)}{N(t_0)} \right\rangle_{t_0}, \quad \text{(S1)}$$

where $\langle \cdots \rangle_{t_0}$ indicates a weighted average over all valid time origins t_0, with weights given by $w(t_0) = \frac{N(t_0)}{\sum_{t_0'=0}^{T-1} N(t_0')}$. Thus, $C(\tau)$ is a weighted average of the conditional survival probability

given that a molecule is in the first solvation shell at time t_0, $\frac{S(t_0,t_0+\tau)}{N(t_0)}$, i.e. the fraction of water molecules that remain continuously in the first solvation shell from time t_0 to $t_0+\tau$, weighted by the number of water molecules in the first solvation shell at time t_0. To compute the continuous lifetime, we then integrate the continuous correlation function:

$$\tau_{\mathrm{c}} = \int_0^T C(\tau) d\tau. \tag{S2}$$

Supplementary Figures

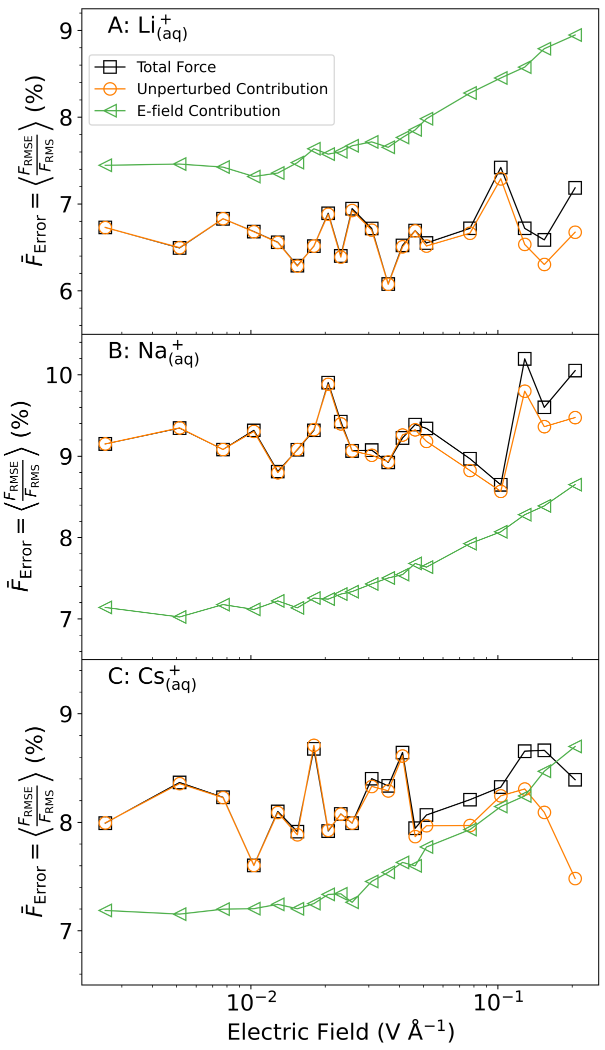

Figure S1: Force prediction score as a function of the electric field strength for the different ions.[2] Panels A, B and C contain results for $Li^+_{(aq)}$, $Na^+_{(aq)}$ and $Cs^+_{(aq)}$, respectively. Black squares, orange circles and green triangles correspond to the score of the total, unperturbed and perturbation induced forces, respectively. The score across all three systems for the total, unperturbed and perturbation induced forces is always below 10% , indicating excellent agreement with the DFT reference data.

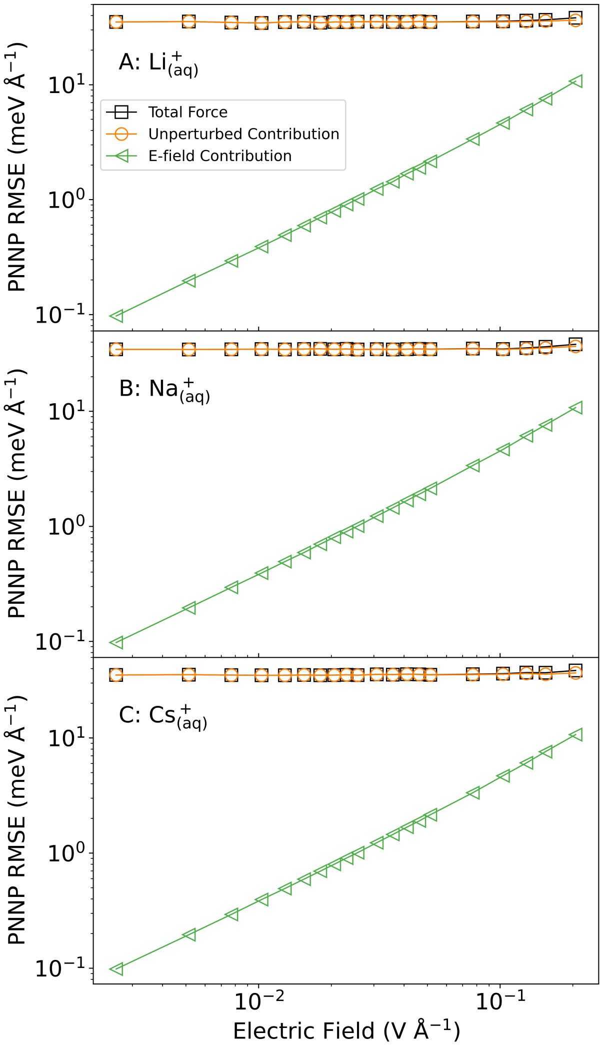

Figure S2: Root-mean-square error (RMSE) of the forces as a function of the electric field strength for the different ions. Panels A, B and C contain results for $Li^+_{(aq)}$, $Na^+_{(aq)}$ and $Cs^+_{(aq)}$, respectively. Black squares, orange circles and green triangles correspond to the RMSE of the total, unperturbed and perturbation induced forces, respectively. The error for all three systems are dominated by the unperturbed forces, which are calculated using a committee of neural network potentials (NNPs).

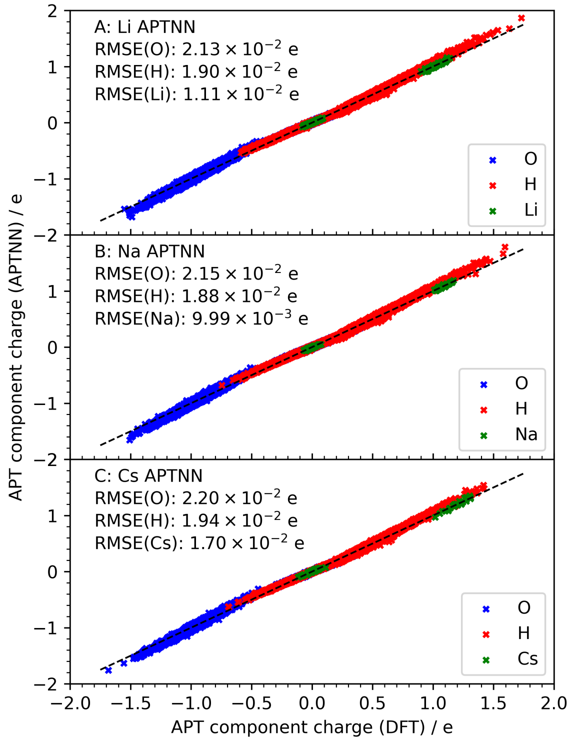

Figure S3: Parity plots of the atomic polar tensor neural network (APTNN) predicted vs. reference DFT values for atomic polar tensor elements. Panel (a) shows results for $Li^+_{(aq)}$, panel (b) for $Na^+_{(aq)}$ and panel (c) for $Cs^+_{(aq)}$.

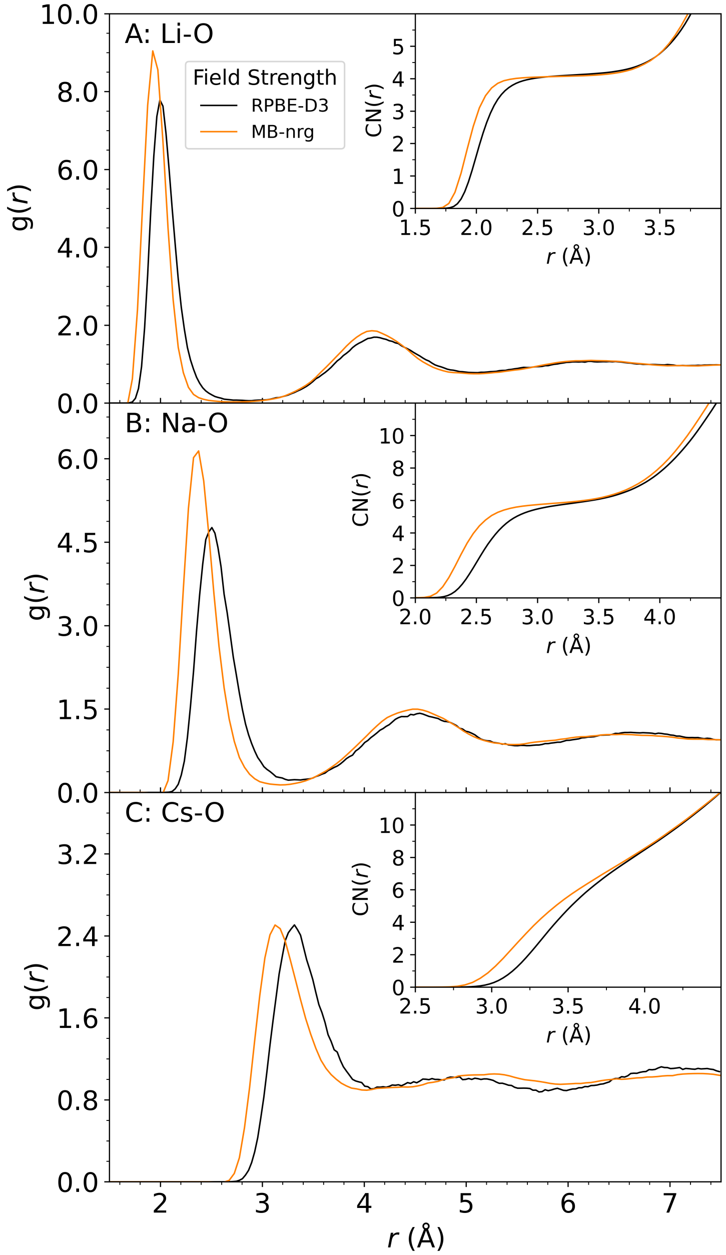

Figure S4: Comparison of the ion radial distribution functions (RDFs) obtained from PNNP MD simulations and those from MB-NRG calculations. Black lines indicate the results obtained from this study and orange lines those from Ref.[3] The RDFs are shown for (a) Li^{+}-O, (b) Na^{+}-O, and (c) Cs^{+}-O pairs. Qualitatively, the RDFs from both methods are in good agreement, with only minor differences in peak heights and positions.

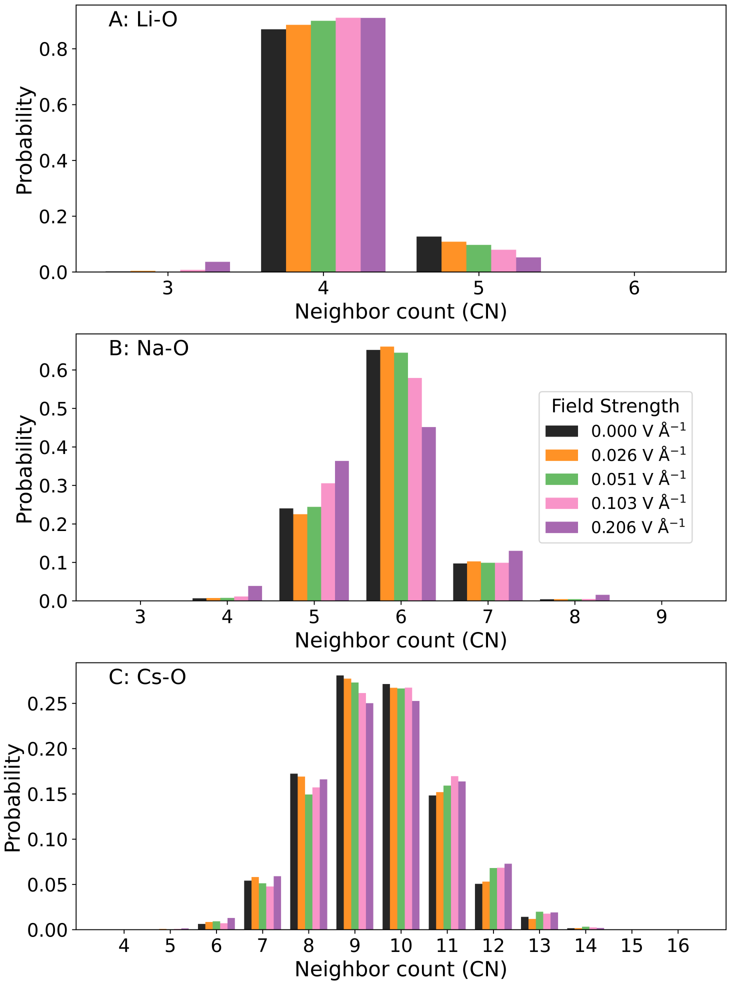

Figure S5: Histograms of the integer coordination numbers at different field strengths for (a) $Li^+_{(aq)}$, (b) $Na^+_{(aq)}$ and (c) $Cs^+_{(aq)}$. The coordination numbers were calculated using the first solvation shell radial cutoff at zero field, displayed by a dashed black line in Fig. 1 and reported in Tab. S1.

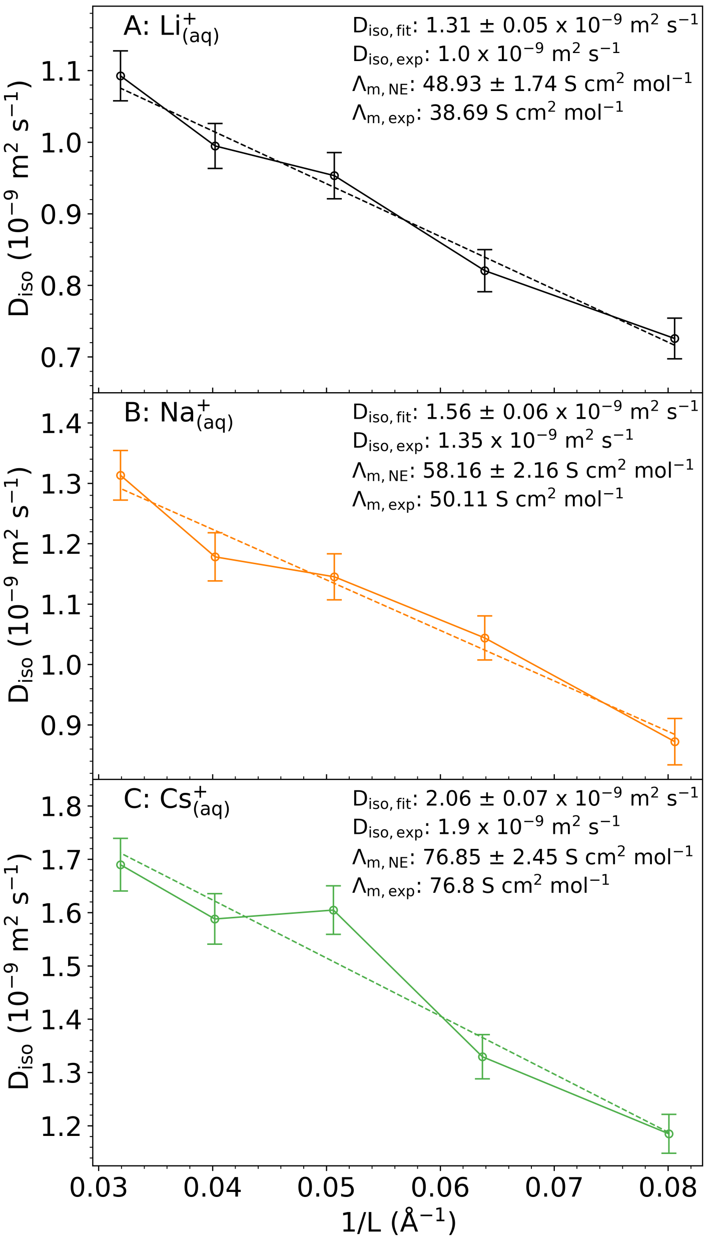

Figure S6: Comparison of the ionic conductivities obtained from zero-field c-NNP MD simulations using the Green-Kubo relation as a function of the 1/L extrapolated to infinite dilution. Panels A, B and C contain results for $Li^+_{(aq)}$, $Na^+_{(aq)}$ and $Cs^+_{(aq)}$, respectively. The ionic conductivities were calculated from the ionic current autocorrelation function using the Green-Kubo relation and the bootstrapping procedure outlined in the *Computational Details* section.[4] The mean and errors reported are the mean and standard deviation of the bootstrapped distribution. The dashed lines are linear regressions to the data points, which were used to extrapolate to infinite dilution ($1/L \rightarrow 0$). The extrapolated values are summarised in Tab. S3.

Supplementary Tables

Table S1: Summary of ion–oxygen RDF features at each field: first peak position r_{max}, first minimum r_{min}, and coordination number at r_{min}.

Ion	Field (V Å^{-1})	r_{max} (Å)	r_{min} (Å)	CN(r_{min})
$Li^+_{(aq)}$	0.00	2.00	2.88	4.13
	0.03	2.00	2.84	4.10
	0.05	2.00	2.84	4.09
	0.10	2.00	2.84	4.07
	0.21	2.00	2.84	4.01
$Na^+_{(aq)}$	0.00	2.51	3.33	5.86
	0.03	2.51	3.35	5.91
	0.05	2.51	3.35	5.89
	0.10	2.51	3.32	5.79
	0.21	2.48	3.32	5.73
$Cs^+_{(aq)}$	0.00	3.32	4.18	9.25
	0.03	3.32	4.10	9.04
	0.05	3.32	4.13	9.36
	0.10	3.32	4.34	10.88
	0.21	3.29	4.58	12.67

Table S2: Continuous first-shell water-ligand lifetimes τ_c (ps) from the survival correlation function at different fields.

Ion	Field ($V\,Å^{-1}$)	τ_c (ps)
$Li^+_{(aq)}$	0.000	20.5
	0.026	26.8
	0.051	20.5
	0.103	22.5
	0.206	14.3
$Na^+_{(aq)}$	0.000	5.8
	0.026	6.8
	0.051	6.4
	0.103	6.5
	0.206	2.8
$Cs^+_{(aq)}$	0.000	2.2
	0.026	2.1
	0.051	2.1
	0.103	2.5
	0.206	2.0

Table S3: Molar ionic conductivity Λ_m from finite-field regression compared to Green–Kubo and experiment ($S\,cm^2\,mol^{-1}$).

Ion	Λ_m (PNNP MD)	Λ_m (Green-Kubo from 0-field MD)	Λ_m (Exp.)
$Li^+_{(aq)}$	45.7 ± 6.4	48.9 ± 1.7	38.7
$Na^+_{(aq)}$	61.1 ± 5.8	58.2 ± 2.2	50.1
$Cs^+_{(aq)}$	73.9 ± 4.6	76.9 ± 2.5	76.8

Table S4: Parameters for the cubic unit cells used for c-NNP MD simulations made with water density of 0.99659 kg L^{-1} prior to ion insertion.

Ion	Number of water molecules	Cell Length (Å)
$Li^+_{(aq)}$	64	12.409
$Li^+_{(aq)}$	128	15.648
$Li^+_{(aq)}$	256	19.725
$Li^+_{(aq)}$	512	24.857
$Li^+_{(aq)}$	1024	31.322
$Na^+_{(aq)}$	64	12.408
$Na^+_{(aq)}$	128	15.648
$Na^+_{(aq)}$	256	19.724
$Na^+_{(aq)}$	512	24.857
$Na^+_{(aq)}$	1024	31.321
$Cs^+_{(aq)}$	64	12.488
$Cs^+_{(aq)}$	128	15.698
$Cs^+_{(aq)}$	256	19.756
$Cs^+_{(aq)}$	512	24.877
$Cs^+_{(aq)}$	1024	31.334

Table S5: Lowest maximum intensity wavenumber, $\tilde{\nu}$, and the corresponding rattling lifetime, τ_{rattling}, for each ion at zero-field.

Ion	$\tilde{\nu}$ (cm^{-1})	Rattling time period (fs)
$Li^+_{(aq)}$	358.7	93
$Na^+_{(aq)}$	86.6	385
$Cs^+_{(aq)}$	20.0	1667

References

(1) Rapaport, D. Hydrogen bonds in water: Network organization and lifetimes. *Mol. Phys.* **1983**, *50*, 1151–1162.

(2) Schran, C.; Thiemann, F. L.; Rowe, P.; Müller, E. A.; Marsalek, O.; Michaelides, A. Machine learning potentials for complex aqueous systems made simple. *Proc. Natl. Acad. Sci. U.S.A.* **2021**, *118*, e2110077118.

(3) Savoj, R.; Agnew, H.; Zhou, R.; Paesani, F. Molecular Insights into the Influence of Ions on the Water Structure. I. Alkali Metal Ions in Solution. *J. Phys. Chem. B* **2024**, *128*, 1953–1962.

(4) Maginn, E. J.; Messerly, R. A.; Carlson, D. J.; Roe, D. R.; Elliot, J. R. Best practices for computing transport properties 1. Self-diffusivity and viscosity from equilibrium molecular dynamics [article v1. 0]. *Living J. Comput. Mol. Sci.* **2019**, *1*, 6324.
